# Improved Scheme for Data Aggregation of Distributed Oracle for Intelligent Internet of Things

**DOI:** 10.3390/s24175625

**Published:** 2024-08-30

**Authors:** Ruiyang Gao, Yongtao Xue, Wei Wang, Yin Lu, Guan Gui, Shimin Xu

**Affiliations:** 1 Bell Honors School, Nanjing University of Posts and Telecommunications, Nanjing 210003, China; q21010218@njupt.edu.cn; 2 School of Communication and Information Engineering, Nanjing University of Posts and Telecommunications, Nanjing 210003, China; 1020010316@njupt.edu.cn (Y.X.); guiguan@njupt.edu.cn (G.G.); 3 School of Internet of Things, Nanjing University of Posts and Telecommunications, Nanjing 210003, China; b20031209@njupt.edu.cn; 4 Zhongdian Zhiheng Information Technology Service Co., Ltd., Nanjing 210006, China; xushimin.js@chinatelecom.cn

**Keywords:** blockchain, data privacy, homomorphic encryption, Internet of Things, Oracle

## Abstract

Oracle is a data supply mechanism that provides real-world data for blockchain. It serves as a bridge between blockchain and the IoT world, playing a crucial role in solving problems such as data sharing and device management in the IoT field. The main challenge at this stage is determining how to achieve data privacy protection in distributed Oracle machines to safeguard the value hidden in data on the blockchain. In this paper, we propose an improved scheme for distributed Oracle data aggregation based on Paillier encryption algorithm, which achieves end-to-end data privacy protection from devices to users. To address the issue of dishonest distributed Oracle machines running out of funds, we have designed an algorithm called PICA (Paillier-based InChain Aggregation). Based on the aggregation on the Chainlink chain and the Paillier encryption algorithm, random numbers are introduced to avoid the problem of dishonest Oracle machines running out of funds. We use the traffic coverage method to solve the problem of exposed request paths in distributed Oracle machines. Simulation and experimental results show that in small and medium-sized IoT application scenarios with 10,000 data nodes, each additional false request in a single request will result in a delay of about 2 s in data acquisition and can achieve a request response time of 20 s. The proposed method can achieve user data privacy protection.

## 1. Introduction

In recent years, the development of blockchain has gradually expanded into the realm of the Internet of Things (IoT), making significant impacts in fields such as supply chain and healthcare. These applications are all developed based on permissioned blockchains, where participating roles undergo rigorous authentication. For instance, in supply chain scenarios, data uploads are typically restricted to authorized upstream and downstream production units, while in healthcare, data uploads are generally limited to licensed medical organizations and regulatory bodies. However, in potential future application areas such as environmental measurement, where multiple non-authenticated parties may participate, ensuring data reliability, credibility, and privacy becomes a pressing issue that demands immediate solutions.

IoT, the product of the development of new-generation information technology, is also known as Industry 4.0. From smart homes to smart cities, IoT covers virtually every corner of the city [1]. Current IoT systems often rely on a centralized architecture, where all terminals with limited performance resources rely primarily on cloud servers to achieve large-scale computing and storage [2]. Once a server node is attacked, the entire system will collapse. Fortunately, the emergence of blockchain offers a potential solution to the above problems.

Blockchain is a distributed data structure that does not rely on centralized management by a single node. From cloud-based data storage and computing the construction of data transmission relay layers to device connections at the perception layer, academia has provided various solutions from top to bottom for the integration of IoT systems with blockchain [3]. For instance, Wang et al. proposed a hierarchical blockchain storage structure, where the majority of the blockchain is stored in the cloud, while the latest blocks are stored in overlay networks across various IoT devices [4]. Gonçalves et al. developed a file storage solution based on IPFS, and experimental results demonstrated that their solution offers shorter retrieval times compared to direct storage on Ethereum [5]. Dorrid et al. introduced a lightweight blockchain tailored for IoT. They aimed to eliminate the overhead of traditional blockchains while retaining most of their security and privacy advantages [6]. However, this solution introduced centralized management. Addressing issues such as high computational costs and delays in traditional blockchains, Premkumar et al. presented a novel, lightweight, adaptive blockchain and a time-based propagation protocol. Furthermore, they proposed a secure private blockchain system for scenarios that rely on trusted third parties [7].

The data sources for smart contracts on blockchains are Oracles. Acting as a data supply mechanism that provides real-world data to blockchain smart contracts, Oracles played a pivotal role in the explosion of blockchain-based digital currencies from 2020 to 2022. In the early days, to extend the use of blockchains beyond the realm of cryptocurrencies, some researchers and developers considered compromising on decentralization to some extent in exchange for greater interoperability [8]. Existing interoperability technologies have successfully provided the necessary interoperability between various blockchain platforms, but they all have certain limitations. Oracle solutions, being independent of blockchain systems, can overcome most of the limitations of interoperability technologies, thereby facilitating the adoption of Oracle solutions [9]. Academia has also proposed numerous interoperability schemes based on Oracle solutions. For instance, Gao et al. constructed a cross-chain data migration architecture based on Oracles [10]. Sober et al. introduced a blockchain interoperability Oracle based on threshold signatures and voting [11]. Wang et al. presented an extended smart contract system based on Oracle mechanisms and provided a reward and punishment calculation scheme for incentive mechanisms [12].

Existing Oracle systems incorporate cryptography, trusted execution environments, voting mechanisms, and other approaches to provide data to blockchains [13]. For example, addressing the communication between heterogeneous blockchains, Nelaturu et al. proposed a decentralized binary data query Oracle based on voting [14]. Ellis et al. introduced the first decentralized Oracle network built upon Byzantine Fault Tolerance algorithms. To address blockchain network congestion, they further proposed an off-chain aggregation algorithm based on Schnorr threshold signatures [15]. Consequently, some scholars have conducted classified discussions on existing Oracles. Addressing the difficulty in comparing different Oracles, Heiss et al. provided a three-attribute comparison framework focusing on security, liveness, and trustworthiness. They also conducted in-depth evaluations and comparisons of cutting-edge Oracle systems [16]. Bartholic et al. developed a detailed Oracle classification method covering all types of issues. Through this classification, they demonstrated the rationality of the operation of existing Oracle mechanisms [17].

In recent years, blockchain Oracles have gradually emerged as a research hotspot. Many scholars have conducted studies on issues such as Oracle throughput and accuracy. For instance, Gao et al. proposed a new Oracle design pattern that achieves low operational costs and high processing speeds by selecting storage methods without compromising security [18]. Goswami et al. presented a middleware design based on Chainlink to select the optimal data provider within the Chainlink Oracle network [19]. Addressing Oracle’s credibility, AlmiAni et al. constructed a novel graph-based analysis method. This method uses Oracles as nodes and the accumulated average difference in data validity and accuracy as edge weights to distinguish between trustworthy Oracles. Experimental results show that their proposed method achieves approximately 93% accuracy in identifying the credibility of data sources [20].

For IoT applications, Moudoud et al. proposed a scalable blockchain architecture that leverages blockchain sharding and Oracles to establish trust among unreliable IoT devices in a fully distributed manner. Specifically, a P2P Oracle network is employed to ensure the authenticity of data retrieved by IoT devices [21]. Kong et al. introduced a three-tier permissioned blockchain architecture featuring IoT Oracles, where IoT devices in the infrastructure layer act as Oracles for the blockchain, providing real-world data through trusted application interfaces and modular building components [22]. Lin et al. addressed the limitation of blockchains in handling complex smart IoT computing scenarios for digital twins by proposing an architecture that combines computational Oracles with decentralized learning [23].

In the field of cryptography, homomorphic encryption is a type of encryption that allows third parties to perform certain specific computational operations on ciphertext while preserving the original functionality and format characteristics of the ciphertext [24]. Homomorphic encryption is applied both in fog node gateways to prevent the leakage of fog node data and in cloud data security, providing end-to-end security for big data analytics [25,26]. Additionally, homomorphic encryption has gained significant attention in the smart grid domain. For instance, Qiu et al. proposed a dynamic privacy protection scheme based on multi-identity homomorphic encryption, which is not limited to a single key; each user can generate their own key based on their identity [27]. Ma et al. employed the Paillier homomorphic encryption algorithm and a role-based access control policy to ensure privacy security during multi-dimensional aggregation, transmission, and sharing of power data. They incorporated blockchain technology into the smart grid to address privacy and security issues related to power data transmission and sharing [28]. Lu et al. presented a privacy-preserving aggregation scheme for smart grids. They utilized superposition sequences to construct multi-dimensional data and encrypted structured data locally at gateways using Paillier homomorphic encryption [29]. Kang et al. employed an improved identity-based signature algorithm and Paillier homomorphic encryption to protect user privacy. They also used superposition sequences, allowing a single message to report multiple types of data [30]. Karampour et al. utilized network masks from anonymous voting protocols and the Paillier encryption system to protect user data privacy [31]. Chen et al. similarly used the Paillier encryption system, enabling utility providers to obtain only the total consumption of all electricity meters without accessing individual meter records, thereby protecting data privacy [32].

In summary, academic research on Oracles and the application of homomorphic encryption is becoming increasingly popular. However, there is currently a scarcity of studies focusing on data privacy scenarios in distributed Oracles. To address this gap, we propose the application of the Paillier homomorphic encryption algorithm to distributed Oracles, encrypting the transmission of data results to achieve privacy protection for the data.

The main contributions of this paper are as follows:

1. An improved scheme for distributed Oracle data aggregation based on the Paillier encryption algorithm is proposed, which achieves the secure acquisition of encrypted privacy data.
2. To address the issue of dishonest distributed Oracles consuming resources without contributing (i.e., “free-riding”), an improved algorithm named PICA (Paillier-based InChain Aggregation) is designed. This algorithm builds upon the aggregation on the Chainlink chain but introduces randomness based on the Paillier encryption algorithm to prevent dishonest Oracles from free-riding. Additionally, to solve the issue of exposing the original request path in distributed Oracles, a traffic obfuscation method is employed.
3. The proposed scheme was tested and deployed using Raspberry Pis as edge devices. Simulation and experimental results show that in a small to medium-sized scenario with 10,000 data nodes, each additional fake request in a single request can lead to approximately 2 s of delay in data acquisition, but request responses can still be achieved within 20 s. The proposed method successfully achieves data privacy protection for users.

This paper is organized into five sections. Section 1 introduces related work and our contributions. Section 2 elaborates on the proposed improvement scheme. Section 3 describes the deployment environment. Section 4 analyzes the proposed improvement scheme. Section 5 concludes this article.

## 2. Improved Scheme for Trusted Data Aggregation

In this section, we first introduce the framework of the improved scheme for trusted data aggregation based on the Paillier encryption algorithm and then present the improved algorithm for trusted data aggregation utilizing the Paillier encryption algorithm.

### 2.1. Scheme Framework

**Figure 1:**
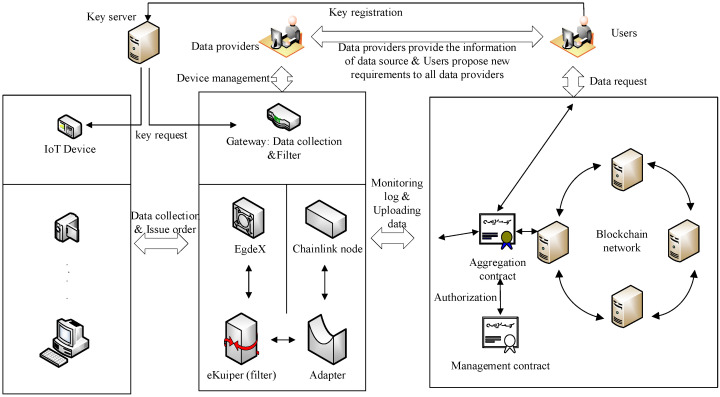
Schematic Diagram of the Improved Framework for Trusted Data Aggregation Based on the Paillier Encryption Algorithm.

Figure 1 illustrates the schematic diagram of the improved framework for trusted data aggregation based on the Paillier encryption algorithm. The architecture comprises six main components: users, data providers, blockchain networks, gateways, IoT devices, and a key server.

Users are the initiators of data requests and the consumption terminals for data. Through interactions with the blockchain network, users can deploy and access private, encrypted data request contracts. Data providers are responsible for maintaining gateway devices and providing users with data source acquisition paths. The blockchain network serves as the primary bearer of various business operations, including running data aggregation mechanisms and developing data value. Gateways are responsible for adapting to heterogeneous and diverse IoT devices downwards and achieving interactions with the blockchain network upwards. They also handle parsing and filtering the data collected by IoT devices. EdgeX and eKuiper are similar to an adapter, where EdgeX connects various devices downward, and eKuiper receives various commands upward. eKuiper can achieve data computation. They transmit data and commands through a publishing/subscribing mechanism. IoT devices represent various sensor-like devices capable of generating and collecting real-time data from the physical world. The public key server is an absolutely trustworthy node that generates a pair of asymmetric keys using the Paillier encryption algorithm. The public key is stored on the server, while the private key is sent to the user through an encrypted channel. The server responds in real-time to public key query requests from IoT devices, gateways, and users on the IoT device side within the framework.

#### 2.1.1. Chainlink Oracle

Chainlink Oracle is the industry-leading decentralized Oracle network that automatically provides real-world data to blockchains. It utilizes distributed data sources and Oracle nodes to build an Oracle network, enhancing the credibility of the data.

Chainlink achieves trustworthy data provisioning through Algorithm 1. This algorithm assumes a total of *n* Oracle nodes, among which *f* are dishonest Oracles, and they satisfy the following relationship:

(1) 3f+1<n

From Algorithm 1, it can be understood that if there are a total of 3*f* + 1 nodes, with at most *f* nodes being dishonest, then at least 2*f* + 1 nodes will submit results in Step 4. In the worst-case scenario, this set of 2*f* + 1 nodes includes *f* dishonest Oracles. Therefore, at least *f* + 1 pieces of data originate from honest nodes.
**Algorithm 1.** On-chain Aggregation AlgorithmWait for the user’s request.Upon receiving the user’s request, assign a unique identifier *sid*.Broadcast (*request*, *sid*).Wait to receive a set C of 2*f* + 1 messages from *O*_i_ (commit, *c*_i_ = Commitri(*A*_i_), *sid*).Broadcast (committed, *sid*).Wait to receive a set D of *f* + 1 valid revelation results, where all elements *A*_i_ in the set equal a certain value *A*.Send (*Answer*, *A*) to users.

#### 2.1.2. Paillier Encryption Algorithm

The Paillier algorithm is an additive homomorphic encryption algorithm based on the composite residuosity problem. It introduces a new probabilistic encryption scheme that permits addition operations to be performed on encrypted data, and the decrypted result will accurately reflect the sum of the original values of those encrypted data.

(2) $$x^{n}\equiv a(mod\;n^{2})$$

The key generation algorithm is as follows: for large prime numbers *p* and *q*,

(3) gcd(pq,(p−1)(q−1))=1

where *gcd* is the greatest common divisor function.

Calculate the product number *n* and the greatest common divisor *λ*:

(4) n=pq

(5) λ=lcm(p−1,q−1)

where *lcm* is the least common multiple function.

Select a random integer $g\in {\mathbb{Z}}_{n^{2}}^{*}$, where ${\mathbb{Z}}_{n^{2}}^{*}$ is an integer multiplication subgroup of modulo *n*^2^. Try

(6) $$\gcd(n,L(g^{\lambda \mathrm{mod}n^{2}}))=1$$

where function *L* is defined as

(7) L(u)=(u−1)/n

(8) $$\mu =L{\left(g^{\lambda }\;mod\;n^{2}\right)}^{-1}\;mod\;n$$

The variable *u* in the subgroup ${\mathbb{Z}}_{n^{2}}^{*}$ is a multiplicative subgroup of integer modulo *n*^2^. The public key obtained is (*n*, *g*), and the private key obtained is (*λ*, *μ*).

The encryption algorithm is as follows: for plaintext *m* and random number *r*, perform the following calculations.

(9) $$c=E(m)=g^{m}r^{n}(mod\;n^{2})$$

among them, *c* is the ciphertext, and *E* is the homomorphic encryption function.

The decryption algorithm is as follows: for ciphertext *c* < *n*^2^, calculate

(10) $$D(c)=\frac{L(c^{\lambda }(mod\;n^{2}))}{L(g^{\lambda }(mod\;n^{2}))}mod\;n=m$$

Among them, *D* is the homomorphic decryption function.

(11) $$E(m_{1})*E(m_{2})=(g^{m_{1}}r_{1}^{n}(mod\;n^{2}))*(g^{m_{2}}r_{2}^{n}(mod\;n^{2}))$$

(12) $$=g^{m_{1}+m_{2}}{\left(r_{1}*r_{2}\right)}^{n}(mod\;n^{2})=E(m_{1}+m_{2})$$

### 2.2. PICA Algorithm

Algorithm 2 is a privacy-preserving data aggregation algorithm PICA based on Paillier encryption designed in this paper.
**Algorithm 2.** PICA Privacy Protection AlgorithmWait for user data requests.Receive a user request with a unique identifier *sid*.Broadcast (*request*, *sid*).Wait for the ciphertext set C (*Encrypt*(*A*_i_, *rand*), *sid*) to receive 2*f* + 1 messages from *O*_i_.Broadcast (*resend*, *sid*).Wait to receive *f* + 1 ciphertext sets D and their corresponding *rand*; All ciphertexts in the collection are stored in the contract.

The user obtains the ciphertext set D from the contract, decrypts it using Paillier, and verifies the legitimacy of the set result with a random integer rand, and finally takes the median. Among them, *sid* represents the session identifier *ID* requested by the user, *O_i_* represents the *i*-th Oracle, Encrypt represents the encryption operation, specifically referring to the Paillier encryption algorithm, and Ai represents the result of the *i*-th Oracle.

Distributed Oracles often suffer from the issue of freeloading, where dishonest Oracle nodes steal and replicate the results from honest Oracle nodes without contributing to the computation. Chainlink’s proposed Algorithm 1 is a straightforward sequential protocol that ensures protocol availability with 3*f* + 1 nodes. It prevents freeloading behavior through a commit-then-reveal mechanism. Algorithm 2 presented in this paper also guarantees protocol availability with 3*f* + 1 nodes, but to avoid freeloading by dishonest Oracle nodes, it employs the Paillier encryption scheme, leveraging its unique properties as follows:

Homomorphic scalar multiplication property: for ciphertext *c*_1_ and scalar *rand*, calculate

(13) $$c=c_{1}^{rand}\;mod\;n^{2}$$

which obtains ciphertext *c*.

Homomorphic scalar multiplication property verification: Decrypt ciphertext *c* to obtain the product of plaintext *m*_1_ and scalar *rand*:

(14) $$D(c_{1}^{rand}\;mod\;n^{2})=D(g^{m_{1}rand}r^{n}\;mod\;n^{2})\;=m_{1}rand$$

The algorithm proposed in this paper, after the Oracle nodes obtain the ciphertexts, performs homomorphic scalar multiplication with a random number *rand*. Through two rounds of communication, a set of ciphertexts D containing *f* + 1 results and their corresponding random values *rand* are obtained. Similar to the principle of Algorithm 1, by first committing to the results and then revealing the decrypted random values, the algorithm prevents freeloading behavior from occurring.

### 2.3. Command Mapping

**Figure 2:**
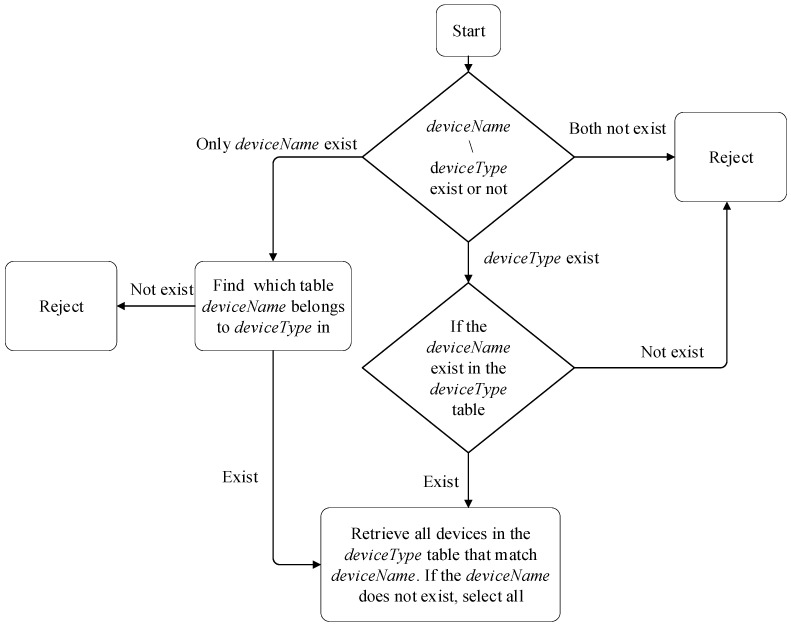
Device Command Mapping Process.

After receiving the command, Oracle needs to interact with the perception layer. To achieve automated processes, we design a command mapping algorithm flow, as shown in Figure 2.

The execution process of the algorithm flow is as follows:

1. Parse the command packet to determine if there is *deviceName* or *deviceType* in the request. If none of them exist, discard them directly.
2. If only *deviceName* exists in the command packet, search for the *deviceType* belonging to *deviceName*. If there is no local ownership table, discard it directly.
3. If the command packet contains *deviceType*, verify if the local ownership table exists. If it does not exist, discard it directly.
4. If there is a *deviceType* attribution table locally, further verify if there is *deviceName* in the table. If it does not exist, discard it directly.
5. Read all devices in the *deviceType* table that match the *deviceName*. If it does not exist, select all.

### 2.4. Blockchain-Based Workflow for IoT Oracle

**Figure 3:**
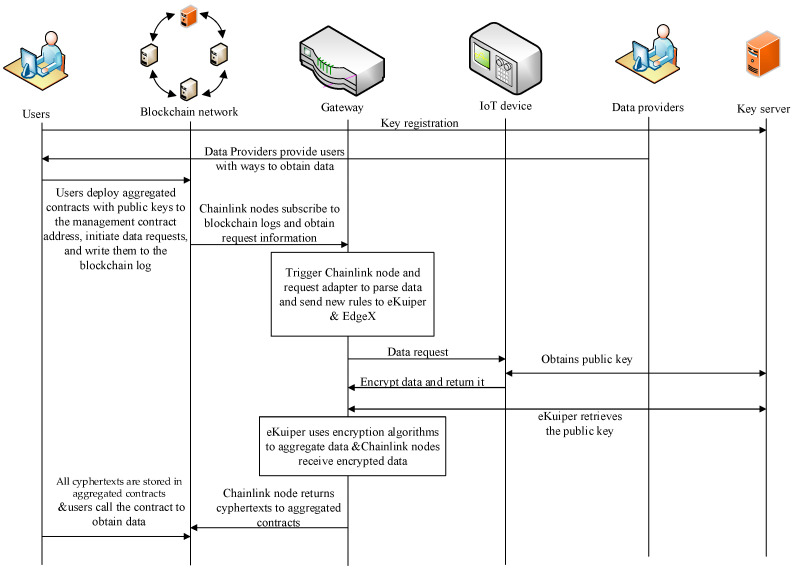
The Workflow of the Proposed Scheme.

This section introduces the workflow of the proposed solution, as shown in Figure 3.

1. Users use their *ID* to register key pairs on the key service based on the Paillier encryption algorithm.
2. Data providers provide users with on-chain data acquisition methods. Users deploy their own aggregation contract and preset their public key in the contract, calling the contract request function.
3. Chainlink nodes read logs from the blockchain and send request packets to eKuiper.
4. eKuiper and EdgeX collaborate to generate device commands.
5. The IoT device receives a trigger command from EdgeX and accesses the key server to request the public key corresponding to the user *ID*, while collecting data from the device once.
6. After obtaining the public key and data, the IoT device encrypts the data and returns it to EdgeX.
7. eKuiper subscribes to the data from EdgeX, follows the process in Figure 3 to aggregate the data, and returns it to Chainlink.
8. Chainlink converts and sends data to blockchain aggregation contracts.
9. The aggregation contract uses the user’s prestored public key to aggregate data and obtains the encrypted final value.
10. Users choose to process the ciphertext themselves or a trusted third party and ultimately obtain the data content.

## 3. Deployment Environment

This section details the deployment of the proposed scheme. The hardware setup consists of a PC equipped with an Intel(R) Core(TM) i7-7700HQ@2.8GHz processor (manufactured by Intel Corporation, Santa Clara, CA, USA) and 16 GB of RAM, along with Raspberry Pi 4B devices (manufactured by Raspberry Pi Foundation, Cambridge, UK). Chainlink and the key server are run on the PC, while EdgeX, Chainlink adapters, and IoT device adapters are initiated on the Raspberry Pi, which is also connected to a B-TH-RS30 temperature and humidity sensor (manufactured by Lankong Electronic Technology Co., Ltd., Huai’an City, China).

The Goerli Ethereum test network is utilized as the blockchain network, and the Goerli API node provided by Alchemy serves as the blockchain node. Contracts are deployed using the Hardhat and Solidity programming languages. All implementations mentioned below are completed on the Goerli test network.

## 4. Analysis

The purpose of this paper is to establish a data-sharing platform that safeguards user privacy. Data providers maintain the data source equipment for this platform and can receive a certain amount of compensation; that is, data is supplied at a price. Users spend a certain amount of money to obtain data, and data providers receive corresponding rewards. To evaluate the feasibility of the proposed privacy protection scheme, the following aspects need to be considered.

### 4.1. Analysis of System Privacy

Based on the proposed scheme in this paper, we have established a security model. The system involves six key roles: users, data providers, other nodes on the blockchain network, gateway devices, IoT devices, and key servers. In a public environment, potential attackers may include

*(1)*  *Users and other nodes on the blockchain*

Launching a large number of false requests causes overheating of IoT devices or gateway devices, leading to system downtime. This issue can be effectively addressed through a paid data acquisition model, where users are required to pledge a certain amount of currency to obtain data from the blockchain.

Method 2: Launching a DDoS attack against the key server. In our proposed scheme, the key server is not only trusted but also must be reliable, as it plays a crucial role in protecting data privacy.

*(2)*  *Data providers and gateway devices*

Method 1: Sending random and falsified data. According to Byzantine Fault Tolerance principles, this attack can only be effective if inequality (1) does not hold, i.e., when dishonest gateways or data providers constitute more than one-third of the total data providers. This issue can be temporarily mitigated by tracking the historical service levels of data providers.

Method 2: Launching a DDoS attack against IoT devices to cause downtime. However, since IoT devices and gateway devices employ a publish-subscribe data transmission model with the gateway device serving as the server, this method is ineffective.

Method 3: Providing false request paths. This issue can also be temporarily addressed by tracking the historical service levels of data providers.

*(3)*  *IoT devices and key servers*

In our model, IoT devices and key servers are deployed and guaranteed by trusted organizations, thus ensuring their security and reliability.

The proposed scheme in this article is based on blockchain communication and utilizes Chainlink’s InChain protocol. It employs the Paillier encryption algorithm to generate encrypted data for transmission, achieving end-to-end communication security. Therefore, it is necessary to ensure communication security in three aspects:

First, the blockchain network must be operating normally and be resilient to attacks such as Sybil and double-spending attacks. Second, Chainlink’s InChain protocol must be secure, which has been explained in [15]. Here, we provide a brief analysis based on the InChain protocol. Assuming there are a total of *n* Oracle nodes participating in data supply and *f* of them are dishonest, they need to satisfy the relationship expressed in Equation (1). The InChain protocol is based on the Byzantine Fault Tolerance algorithm. In the worst-case scenario analysis, in step 4 of Algorithm 2, a set containing data from 2*f* + 1 Oracle nodes is obtained, which includes data from *f* dishonest Oracle nodes and *f* + 1 honest Oracle nodes. Therefore, by selecting the median from the data array containing 2*f* + 1 elements, it can be ensured that the data definitely come from honest nodes. Step 6 of Algorithm 2 guarantees that at least one honest Oracle node is ready. Thirdly, the Paillier encryption algorithm itself has been proven to be semantically secure, and the introduction of the encryption algorithm does not affect the operation of the protocol.

However, despite the data protection offered by the proposed scheme in the distributed Oracle model, the issue of public data request paths still exists. During the process of data requests, the paths of these requests are fully exposed on the blockchain. Other nodes on the chain can access the data sources requested by the user through the same request method, thereby indirectly gaining insight into the user’s behavior. Of course, the on-chain contract can use private variables to hide the request paths. This method can prevent other nodes on the chain from snooping on the data request paths, but the request paths must inevitably be authorized to the gateway level, i.e., the gateway is made aware of the request paths. In this way, the data sources at the gateway also obtain the data request paths.

A feasible solution is to use appropriate cover traffic [33]. Currently, in distributed anonymous communication systems, cover traffic is the only viable solution for sender anonymity. All systems that can achieve sender anonymity can be attributed to a form of cover traffic, with the core idea being that in a peer-to-peer node system, the actual data request information intended to be obtained is mixed with various other data requests to achieve sender anonymity. Therefore, the sender, based on specific data requests, mixes meaningless requests with actual requests and constructs multiple request contracts to initiate to the Oracle network, achieving sender anonymity. The drawback of the proposed scheme in this paper is that the data request path is exposed and public, allowing snoopers to read the data request path and initiate the same data request to the blockchain, thereby indirectly obtaining the corresponding data. A simple solution is to configure multiple data aggregation contracts or register multiple public keys at the user end, hiding the actual target data request among other invalid requests and encrypting the data with randomly registered public keys. In this way, even if the target data request path is exposed, intruders cannot understand the purpose of the data usage. At the same time, the randomly registered public keys make the *ID* in the request path change randomly, achieving the effect of hiding the purpose of data usage.

### 4.2. Analysis of System Communication Frequency

The introduction of cover traffic is bound to increase the communication load of the system. Below, we conduct a simulation analysis of this scenario. By adding different numbers of false conditions to a single request, we observe the relationship between the communication frequency of the blockchain network and the number of aggregation nodes. The communication frequency of the blockchain network is defined as the number of communications within the blockchain network for a single request. The following data is derived from simulation tests.

**Figure 4:**
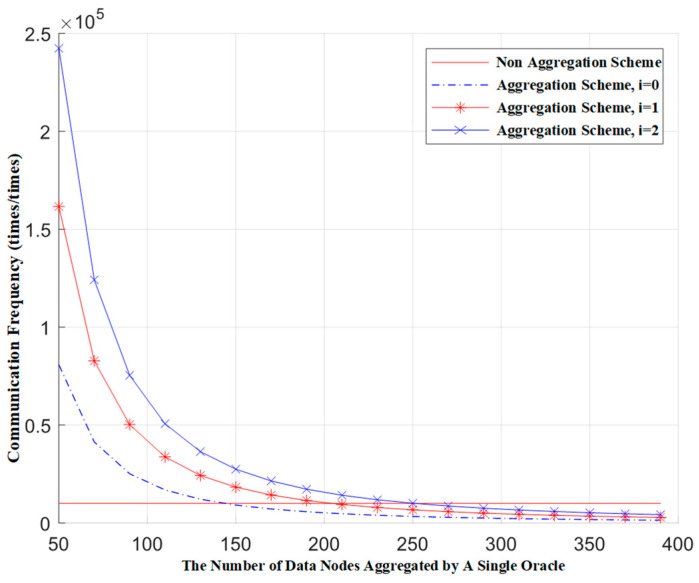
The relationship between the communication frequency of blockchain networks and the number of data nodes aggregated by a single Oracle.

Figure 4 shows the relationship between the communication frequency of the blockchain network and the number of aggregation nodes when different numbers of false requests are mixed in. In the figure, communication frequency represents the number of blockchain network communications corresponding to a single request, and the total number of nodes represents the total number of data nodes across all gateways. Here, the total number of data nodes is 10,000, and *i* represents the number of false requests mixed in. From the figure, we can see that, under the premise of ensuring the same communication frequency for both the aggregation scheme and the non-aggregation scheme, each additional false request in a single request requires the Oracle gateway to aggregate approximately 50 more data source nodes. Furthermore, under the condition that the Oracle gateway aggregates the same number of data source nodes, each additional false request results in an increase in the communication frequency of the blockchain network that is equal to the communication frequency of a single genuine request.

**Figure 5:**
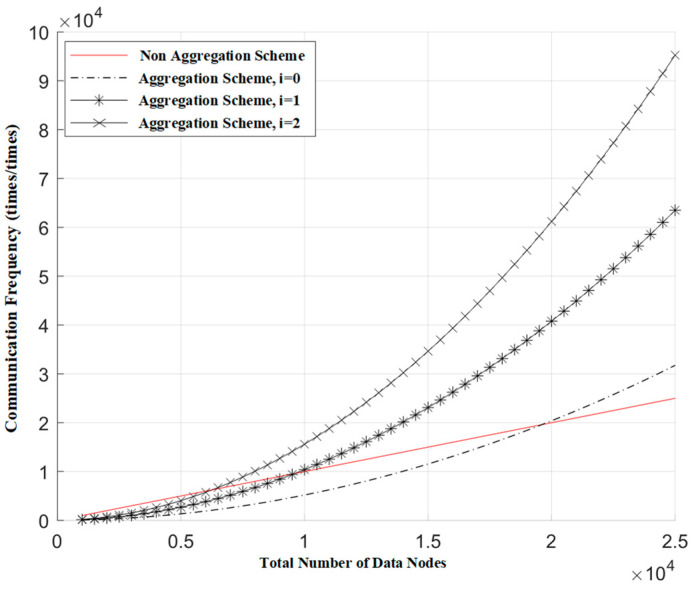
The relationship between the communication frequency of blockchain networks and the total number of data nodes.

Figure 5 illustrates the relationship between the communication frequency of the blockchain network and the total number of data nodes when different numbers of false requests are mixed in. In this figure, communication frequency represents the number of blockchain network communications corresponding to a single request, and the total number of nodes signifies the total data nodes across all gateways. Here, the number of data nodes aggregated by a single Oracle gateway is 200, with a total of 5000 data nodes in the system, and *i* represents the number of false requests mixed in. From the figure, it can be observed that, under the premise of maintaining the same communication frequency as the non-aggregation scheme, for every additional false request, the total number of data source nodes needs to be reduced by approximately half.

### 4.3. Analysis of the Impact of Paillier Algorithm on System Latency

Chainlink’s on-chain contract aggregation scheme addresses the issue of data trustworthiness under decentralized conditions. However, the proposed solution in this paper, which utilizes the Paillier encryption algorithm to conceal plaintext data on top of Chainlink’s on-chain contract aggregation, prevents data sorting within the on-chain aggregation contract, thus failing to guarantee that data is immune to the influence of dishonest data providers. Dishonest providers might inject abnormal or falsified data, even if the original data sources are correct and uncontaminated.

To tackle this issue, we introduce the PICA algorithm. The ciphertext array within the on-chain aggregation contract is made accessible to the contract owner, enabling the owner (user) to manually decrypt it to plaintext after the data is uploaded to the aggregation contract. This allows users to decrypt and verify the data in a trusted environment before aggregation, thereby ensuring the credibility of the data obtained by the proposed solution for data requesters. Nevertheless, this method requires the decrypting party to possess devices with certain performance capabilities, especially when dealing with large amounts of request data, thereby raising the barrier for accessing encrypted data.

We conducted tests on the decryption process of the Paillier encryption algorithm using an Intel(R) Core(TM) i7-7700HQ@2.8GHz quad-core processor (manufactured by Intel Corporation, Santa Clara, CA, USA). The average decryption times are presented in Table 1. The decryption times for different bit sizes of the Paillier encryption algorithm differ by almost an order of magnitude, while different file sizes have little impact on the decryption time. On the equipment used in this paper, the average decryption time for the 256-bit Paillier encryption algorithm is approximately 10,000,000 nanoseconds, or 0.01 s. In a single user request, it is feasible to decrypt ciphertexts from nearly a hundred Oracles simultaneously, with processing speeds sufficient for individual users or small applications.

Compared to RSA and ElGamal algorithms, the Paillier encryption algorithm typically exhibits longer encryption and decryption times, as well as longer ciphertexts. We conducted encryption time tests for the Paillier encryption algorithm using a Raspberry Pi 4B hardware device, with average times listed in Table 2. As can be seen from Table 2, changes in file size within the range of 2 to 64 bits have a noticeable impact on the time consumed by Paillier encryption. The average time for a single 256-bit Paillier encryption operation is approximately 40,000,000 nanoseconds, or 0.04 s, equating to approximately 25 encryptions per second. In the context of smart IoT application scenarios, a single Raspberry Pi 4B device can homomorphically aggregate data from 25 data source nodes per second, which is insufficient for medium to large-scale sensor networks. However, in smaller-scale scenarios, increasing the number of gateways can reduce the number of data nodes that each device needs to aggregate, which is a viable approach.

For instance, in the data privacy analysis of Section 4.1, assuming that each Oracle needs to aggregate 400 data nodes with a sufficiently fast block time and high throughput in the blockchain network, the time required to obtain off-chain data once is no more than 20 s, with each Oracle taking 16 s to read and process data. Additionally, in a scenario with 10,000 data source nodes, each additional false request would require gateways to aggregate an additional 50 data source nodes, adding approximately 2 s of delay per request. Furthermore, with the advancement of hardware devices, the computational capabilities of edge devices are improving exponentially. It is expected that in the future, the computational performance of edge devices will no longer be a limitation for running homomorphic encryption schemes.

## 5. Conclusions

This paper focuses on the research of implementing data privacy protection in distributed Oracles and proposes an improved scheme for distributed Oracle data aggregation based on the Paillier encryption algorithm. We utilize the Paillier encryption algorithm to encrypt data during transmission, which is ultimately decrypted by the user end, thereby achieving user privacy protection. To address the issue of exposing data request paths, we propose an appropriate method of using cover traffic. An IoT device adapter software interface is defined, and the proposed scheme is tested using the Ethereum Goerli testnet. Encryption and decryption time tests for the Paillier encryption algorithm are conducted on both personal computers and Raspberry Pi 4B devices. The simulation and experimental results show that the proposed scheme can effectively protect user data transmission privacy, with a single request result acquisition time delay of less than 20 s.

## Figures and Tables

**Table 1:** Paillier Encryption Algorithm Decryption Average Time.

Key Length	File Size				
	2 Byte	8 Byte	16 Byte	32 Byte	64 Byte
128 bit	4,032,450	4,085,471	4,357,178	4,548,234	4,354,855
256 bit	10,194,568	9,784,532	10,456,874	10,468,156	10,235,408
512 bit	42,565,879	45,684,391	43,254,139	43,225,846	43,581,576
1024 bit	296,081,951	295,558,711	295,543,930	295,481,671	295,700,763

Unit: Nanoseconds.

**Table 2:** Paillier Encryption Algorithm Average Encryption Time.

Key Length	File Size				
	2 Byte	8 Byte	16 Byte	32 Byte	64 Byte
128 bit	7,254,752	7,395,095	7,311,450	7,554,832	7,940,001
256 bit	37,211,149	37,325,481	38,154,832	41,298,200	40,134,089
512 bit	77,147,815	80,351,842	89,147,621	100,167,091	108,248,510
1024 bit	782,415,692	772,458,616	832,478,241	922,468,152	1,002,155,488

Unit: Nanoseconds.

## Data Availability

Data are contained within the article.
